# Investigating the potential mechanism of quercetin against cervical cancer

**DOI:** 10.1007/s12672-023-00788-y

**Published:** 2023-09-13

**Authors:** Man Chu, Huihui Ji, Kehan Li, Hejing Liu, Mengjia Peng, Zhiwei Wang, Xueqiong Zhu

**Affiliations:** Zhejiang Provincial Clinical Research Center for Obstetrics and Gynecology, Department of Obstetrics and Gynecology, The Second Affiliated Hospital of Wenzhou Medical University, Wenzhou Medical University, Wenzhou, 325027 China

**Keywords:** Cervical cancer, Quercetin, Network pharmacology, Treatment, Resistance

## Abstract

**Background:**

Cervical cancer is emerging as a potential target of increased susceptibility to coronavirus disease-2019 (COVID-19), leading to compromised survival rates. Despite this critical link, efficacious anti-cervical cancer/COVID-19 interventions remain limited. Quercetin, known for its efficacy against both cancer and viral infections, holds promise as a therapeutic agent. This study aims to elucidate quercetin’s anti-cervical cancer/COVID-19 mechanisms and potential targets.

**Methods:**

We initiated our investigation with differential gene expression analysis using cervical cancer transcriptome data from The Cancer Genome Atlas (TCGA) and The Genotype-Tissue Expression (GTEx), focusing on intersections with COVID-19-related genes. Network pharmacology was employed to identify the shared targets between cervical cancer/COVID-19 DEGs and quercetin’s targets. Subsequently, Cox proportional hazards analyses were employed to establish a risk score based on these genes. Molecular docking techniques were applied to predict quercetin’s therapeutic targets and mechanisms for mitigating cervical cancer and COVID-19.

**Results:**

Our findings unveiled 45 potential quercetin targets with anti-cervical cancer/COVID-19 actions. Gene Ontology (GO) and Kyoto Encyclopedia of Genes and Genomes (KEGG) analyses highlighted significant enrichment in immune pathways and COVID-19-related pathways. A refined risk score model, comprising PLA2G7, TNF, TYK2, F2, and NRP1, effectively stratified cervical cancer patients into distinct risk groups. Importantly, molecular docking analyses illuminated quercetin’s remarkable binding affinity to the primary protease of the coronavirus.

**Conclusions:**

In summation, our study suggests that quercetin holds promise as a potential therapeutic agent for mitigating coronavirus function, specifically through its interaction with the primary protease. This research offers novel insights into exploring COVID-19 susceptibility and enhancing survival in cervical cancer patients.

## Introduction

Since its emergence in 2019, coronavirus disease-2019 (COVID-19) caused by SARS-CoV-2 has wreaked havoc globally, inflicting profound tolls on public health, economies, and societies [1]. SARS-CoV-2 exhibits various mutants such as Alpha, Beta, Gamma, Kappa, Delta and Omicron [2, 3]. Clinical trials are underway to identify effective treatments for COVID-19. Notably, a randomized controlled trial incorporating lopinavir and ritonavir in severe COVID-19 cases yielded no substantial clinical symptom improvement or reduced mortality compared to standard supportive care [4]. Among the gravest concerns is the heightened susceptibility of cancer patients to COVID-19, resulting in exacerbated symptoms and elevated mortality risk [5]. This predicament is particularly worrisome for cervical squamous cell carcinoma and endocervical adenocarcinoma (CESC) patients, who constitute a significant cohort of cancer cases [6]. Cervical cancer, the fourth most prevalent cancer among women, claims the lives of over 300,000 women annually [7]. According to the statistic, nearly 11 women were dying from cervical cancer every day [8]. The majority of cervical cancer cases are associated with human papillomavirus (HPV) infection. HPV-negative cervical cancer had a worse survival [9]. As a highly vulnerable group, cancer patients are at increased risk for SARS-CoV-2 virus infections due to longer hospital stays and weaknesses in their immune systems [10–12]. COVID-19 infection causes treatment delays and interruption for cervical cancer patients. Given the vulnerability of cancer patients, particularly in the context of the ongoing pandemic, identifying effective therapeutic strategies for cervical cancer patients infected with COVID-19 is imperative.

Quercetin, a polyphenolic flavonoid prominently employed in traditional Chinese medicine, exhibits promising chemo-preventive properties [13, 14]. Its role in modulating signal transduction pathways and augmenting antioxidant defenses has garnered interest in disease prevention [15]. Additionally, quercetin has emerged as an adjuvant in anti-cancer regimens, enhancing treatment sensitivity and minimizing collateral damage to healthy cells [16, 17]. Furthermore, in conjunction with vitamin C, quercetin has demonstrated synergistic potential in augmenting antiviral therapies for COVID-19 [18]. Despite these insights, comprehensive investigations into quercetin’s targets and mechanisms for treating cervical cancer patients with COVID-19 are limited.

**Fig. 1:**
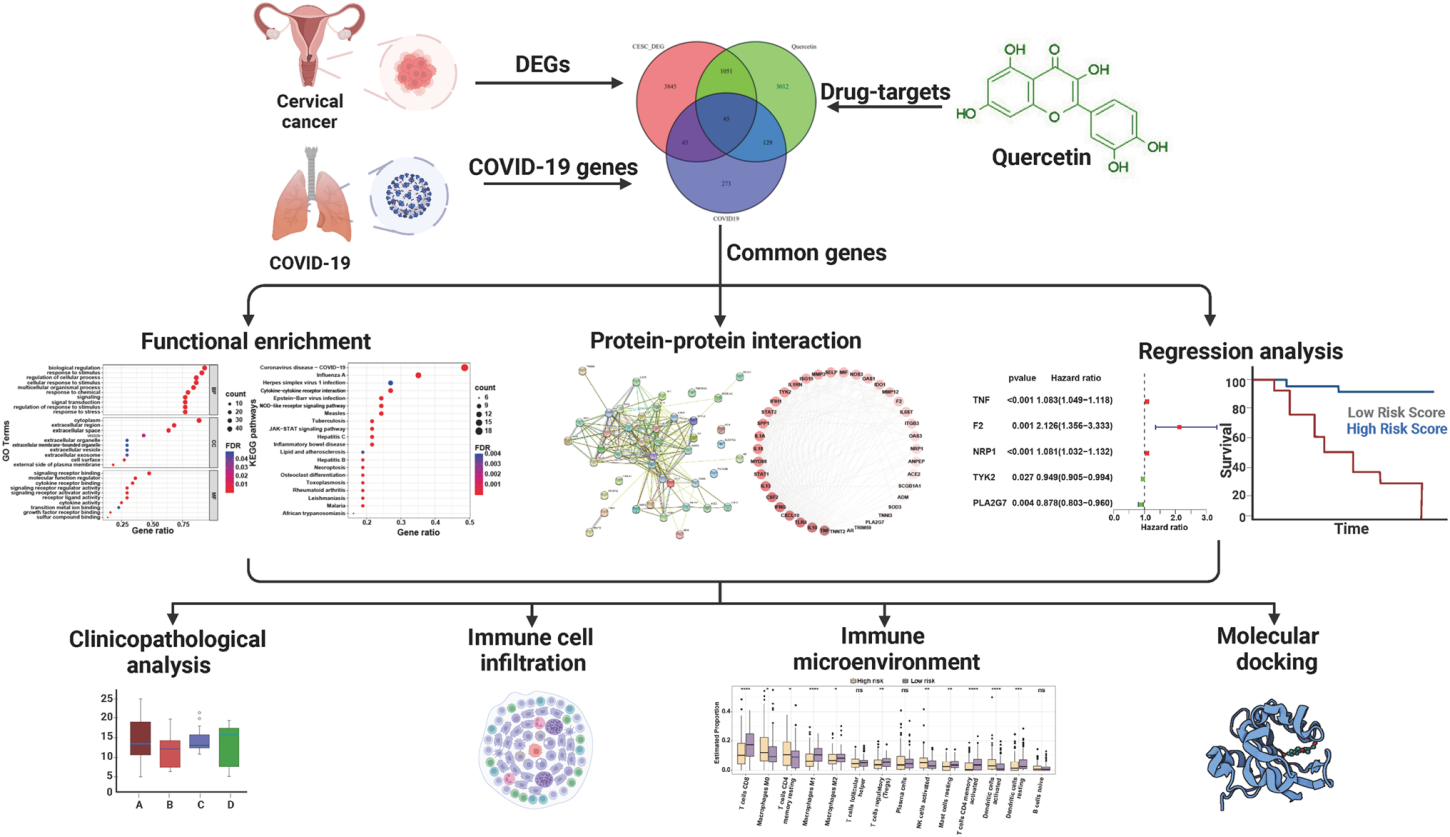
The graphical abstract illustrates the study’s methodology employing network pharmacology and computational bioinformatics to elucidate quercetin’s mechanism against CESC/COVID-19.

In the realm of medicinal research, web-based pharmacology approaches have emerged as robust tools for comprehending the action mechanisms of Chinese medicines [19, 20]. Leveraging network pharmacology, which characterizes drug actions and mechanisms at the molecular level, we aimed to elucidate quercetin’s potential as an adjunctive therapy for cervical cancer patients with COVID-19. The ensuing study delineates our network pharmacology-based analysis, as depicted in Fig. 1.

## Materials and methods

### Screening for core genes associated with COVID-19 and potential differentially expressed genes of CESC

The National Center for Biotechnology Information (NCBI) and the GeneCards Database were employed to identify genes associated with COVID-19 [21]. Transcriptomic profiles of CESC and normal cervical samples were retrieved from The Cancer Genome Atlas (TCGA) on November 05, 2021 Supplementary transcriptomic profiles from the Genotype-Tissue Expression (GTEx) database was included for normal cervical samples [22]. Differential gene expression analysis for CESC was performed using the ‘DEseq2’ package in the R environment, considering genes with false discovery rate (FDR) < 0.05 and |log fold change (FC)| > 1 as differentially expressed. Visualization was facilitated using the ‘EnhancedVolcano’ and ‘ComplexHeatmap’ packages [23, 24]. Overlapping genes between COVID-19-related genes and CESC DEGs were identified.

### Identification of quercetin-related genes and enrichment analysis

Potential quercetin targets were sourced from Comparative Toxicogenomics Database (CTD) [25], DrugBank [26], Swiss Target Prediction [27], TargetNet [28], Kyoto Encyclopedia of Genes and Genomes (KEGG) [29] database. Subsequently, gene ontology (GO) and KEGG enrichment analyses were performed using g:Profiler for enrichment and the ‘ggplot2’ and ‘GOplot’ packages for visualization [30, 31].

### Construction of protein–protein interaction networks

The STRINGv11.5 [32] was employed to construct protein–protein interaction (PPI) networks for quercetin-related genes associated with anti-CESC/COVID-19 effects. Visual representation of results was generated using Cytoscape software (version 3.6.1) [33]. Hub genes were identified using the CytoHubba plugin based on the maximal clique centrality algorithm.

### Clinical analysis for CESC/COVID-19-related genes

Clinical information of CESC patients was obtained from TCGA. Univariate Cox analysis was conducted to identify genes associated with overall survival (OS). Subsequently, multivariate Cox regression analysis were performed using the “glmnet” package to establish a risk score formula based on normalized gene expression values. Patients were categorized into high- or low-risk subgroups using the median risk score. Receiver operating characteristic (ROC) curves (including 3-year, 5-year, and 10-year survival) were plotted to evaluate predictive value using the ‘timeROC’ package.

### Gene set enrichment analysis and immune cell infiltration analysis

Differentially expressed genes between high- and low-risk subgroups were identified using the ‘limma’ package Gene set enrichment analysis (GSEA) was conducted utilizing packages such as “org.Hs.eg.db”, “dplyr”, “enrichplot”, and “clusterProfiler”. Immune cell enrichment scores for 22 immune-related cells were calculated using the CIBERSORT algorithm. Immune cell infiltration content between high- and low-risk subgroups was visualized using the ‘ggpubr’ package. Additionally, the TIMER database [34] was utilized to assess the association between prognostic risk model genes and immune cell infiltration in CESC.

### Prediction of immune checkpoint blockade treatment response

To gauge the risk score’s potential in predicting immune checkpoint blockade treatment response, clinical features and expression data from the IMvigor210 cohort were obtained (http://research-pub.gene.com /IMvigor210CoreBiologies) [35]. The risk score was utilized to predict OS and immune checkpoint blockade therapeutic responses [including progress disease (PD), stable disease (SD), partial remission (PR), and complete remission (CR)].

### Molecular docking

Autodock 4.2 software [36] was employed for semi-flexible molecular docking. Crystal structures of COVID-19 main protease (6LU7) and spike glycoprotein (6VYB) were obtained from the Protein Data Bank (PDB) [37]. The grid box was set to contain all receptor region with the original ligand coordinates. The binding energy was got by autogrid4 and autodock4 function. Molecular docking outcomes were observed using PyMol software [38].

## Result

### Differentiation analysis of CESC

**Fig. 2:**
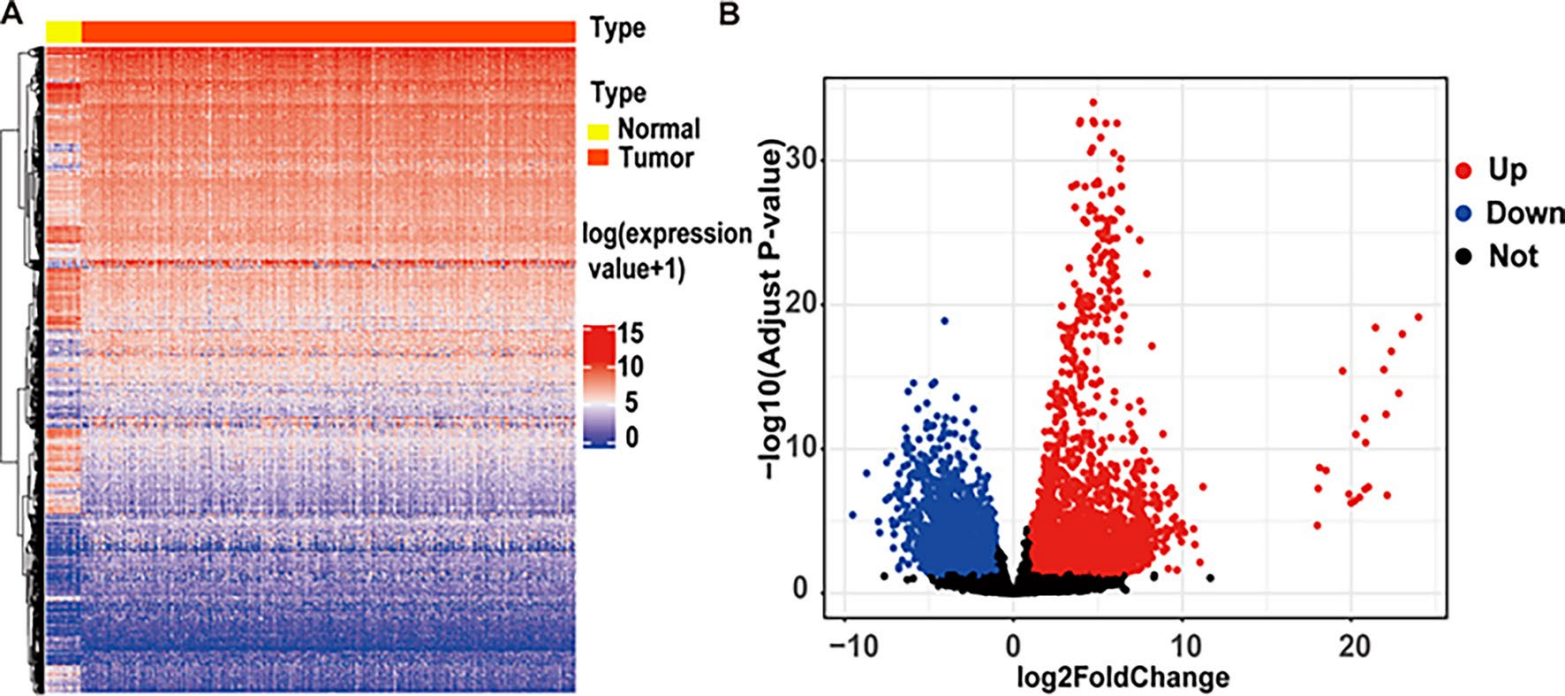
Differential Expression of CESC Genes. **A** Heat map depicting 4986 DEGs in CESC, including 3085 up-regulated and 1901 down-regulated genes. **B** Volcano plot showcasing DEGs in CESC, with red and green dots denoting up-regulated and down-regulated genes, respectively

A comprehensive cohort of 306 CESC samples and 3 normal cervical tissue samples from TCGA, along with 19 cervix samples from GTEx, formed the basis for our investigation. Applying stringent criteria, we identified 4986 differentially expressed genes (DEGs) between CESC patients and controls, with 3085 genes exhibiting up-regulation and 1901 genes down-regulation (Fig. 2).

### Identification of quercetin targets for CESC/COVID-19

**Fig. 3:**
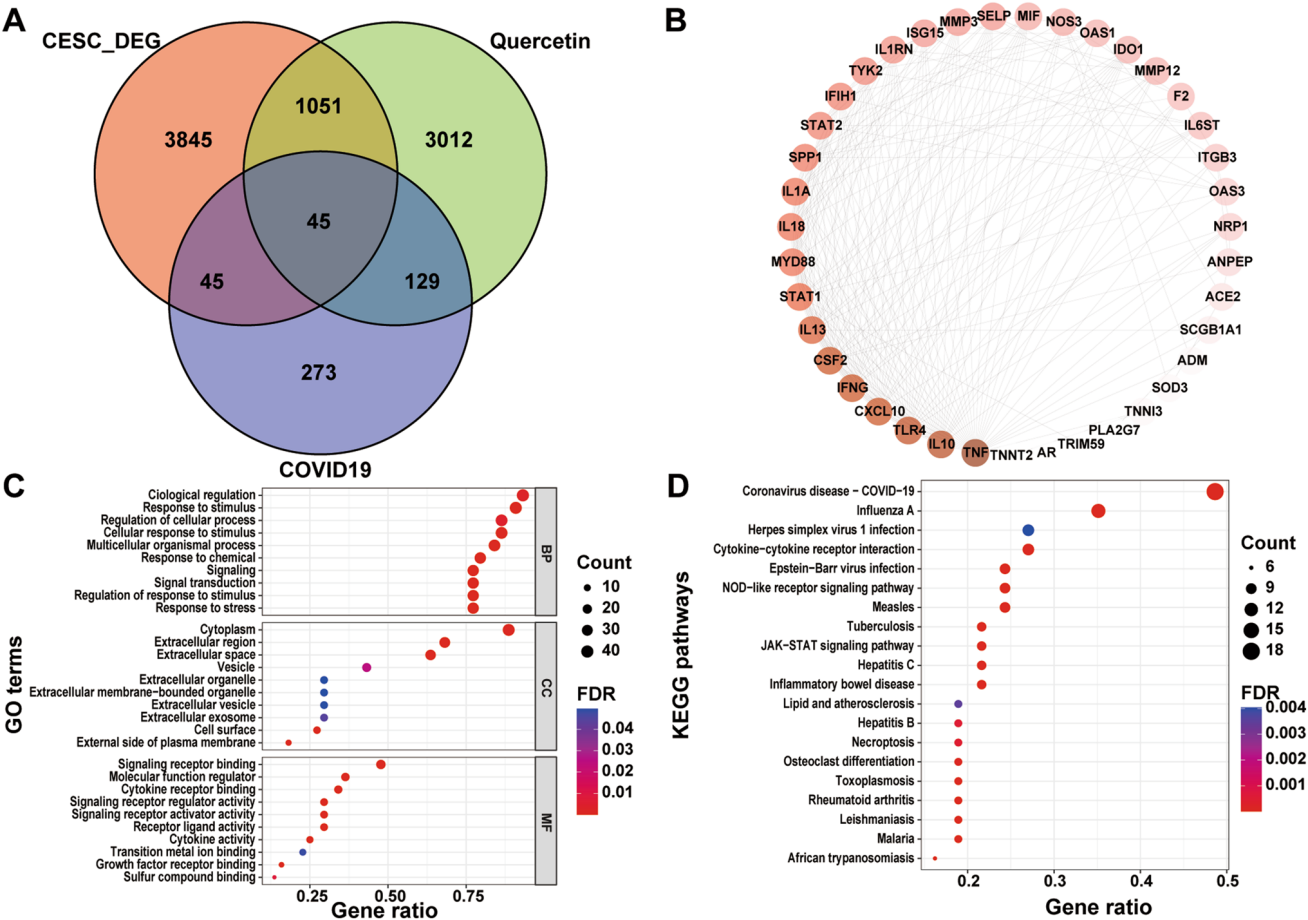
Functional Enrichment Analysis of Quercetin Targets. **A** Venn diagram depicting the intersection of quercetin, CESC, and COVID-19 genes. **B** Protein–protein interaction (PPI) network was visualized using Cytoscape, indicating the gene associations. **C** GO analysis showing the top 10 biological processes (BP), cellular components (CC), and molecular functions (MF) of intersecting genes. **D** KEGG pathway analysis of quercetin targets for CESC/COVID-19.

Incorporating data from NCBI and the Genecard database, we retrieved 492 COVID-19-related genes. A total of 90 CESC/COVID-19-related genes were identified, comprising 62 up-regulated and 28 down-regulated genes in CESC. Further analysis revealed 4237 quercetin-related genes across five databases, including CTD, DrugBank, Swiss Target Prediction, TargetNet and KEGG. Our integration efforts highlighted 45 quercetin targets with implications for CESC/COVID-19, illustrated in Fig. 3A. The resultant PPI network, governed by these 45 intersection genes, was visualized via STRING and Cytoscape (Fig. 3B).

### GO enrichment analysis and KEGG pathway analysis

To illuminate the functional underpinnings of quercetin’s action in the context of CESC/COVID-19, we conducted Gene Ontology (GO) and KEGG pathway analyses on the 45 intersect genes. GO analyses revealed that these genes were predominantly involved in biological regulation, response to stimulus, and cellular response to stimulus. Cellular component enrichment centered around cytoplasm, extracellular region, and extracellular space, while molecular function analysis underscored signaling receptor binding, molecular function regulation, and cytokine receptor binding (Fig. 3C). KEGG pathway analysis unveiled significant enrichment in coronavirus disease-COVID-19, necroptosis, cytokine-cytokine receptor interaction, viral protein interaction with cytokine and cytokine receptor, and an array of signaling pathways (Fig. 3D).

### Independent prognostic signatures and risk analysis

**Fig. 4:**
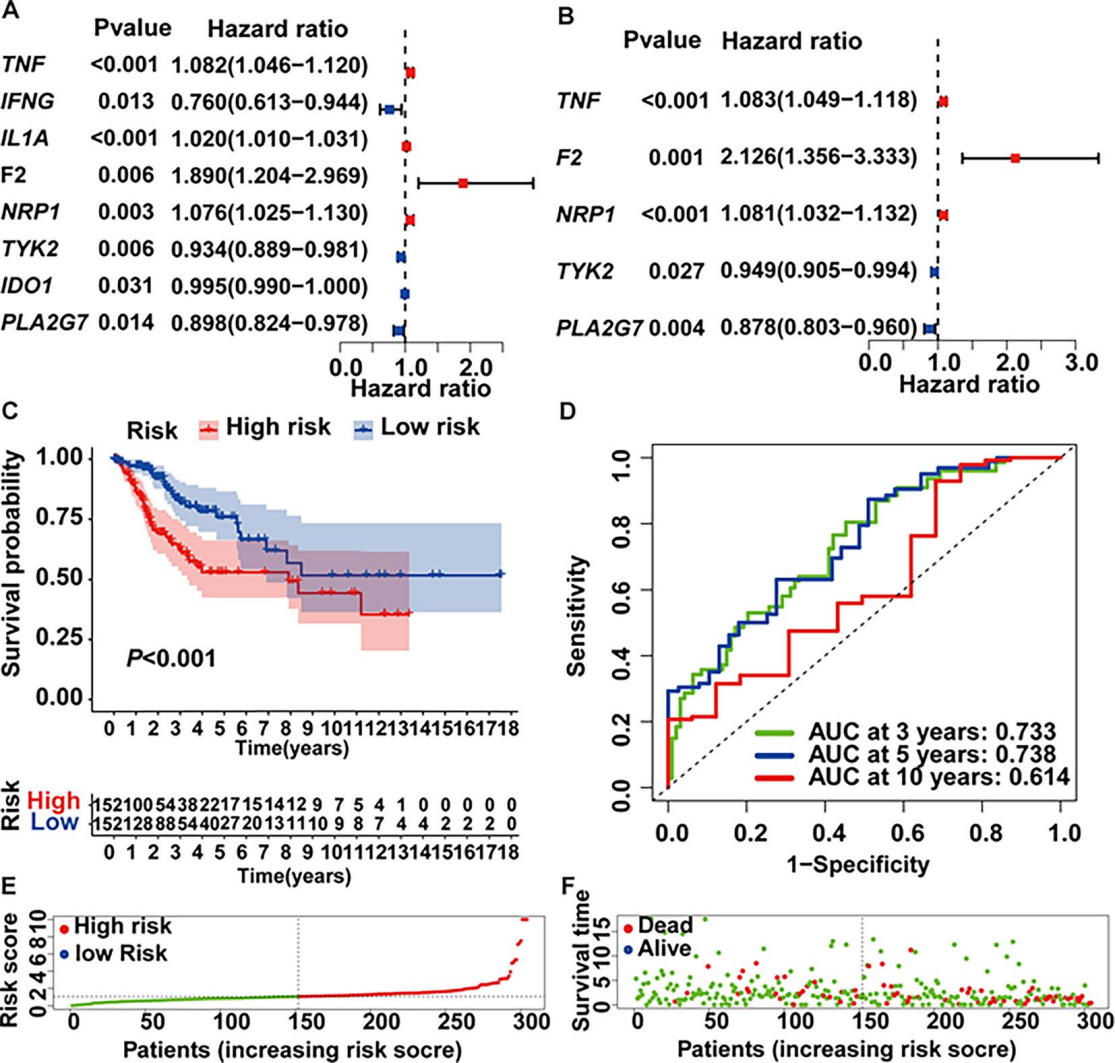
Construction and validation of prognostic signatures. **A** Forest plot presenting HR and P-values from univariate Cox regression analysis. **B** Multivariate Cox analysis revealing independent prognostic signatures. **C** Kaplan-Meier survival analysis indicated significantly shorter OS in the high-risk group (P < 0.001). **D** Time-dependent ROC curves assessing prognostic signature accuracy. **E** Risk score distribution among CESC patients. **F** Scatterplot depicting the correlation between risk scores and survival time/status

**Fig. 5:**
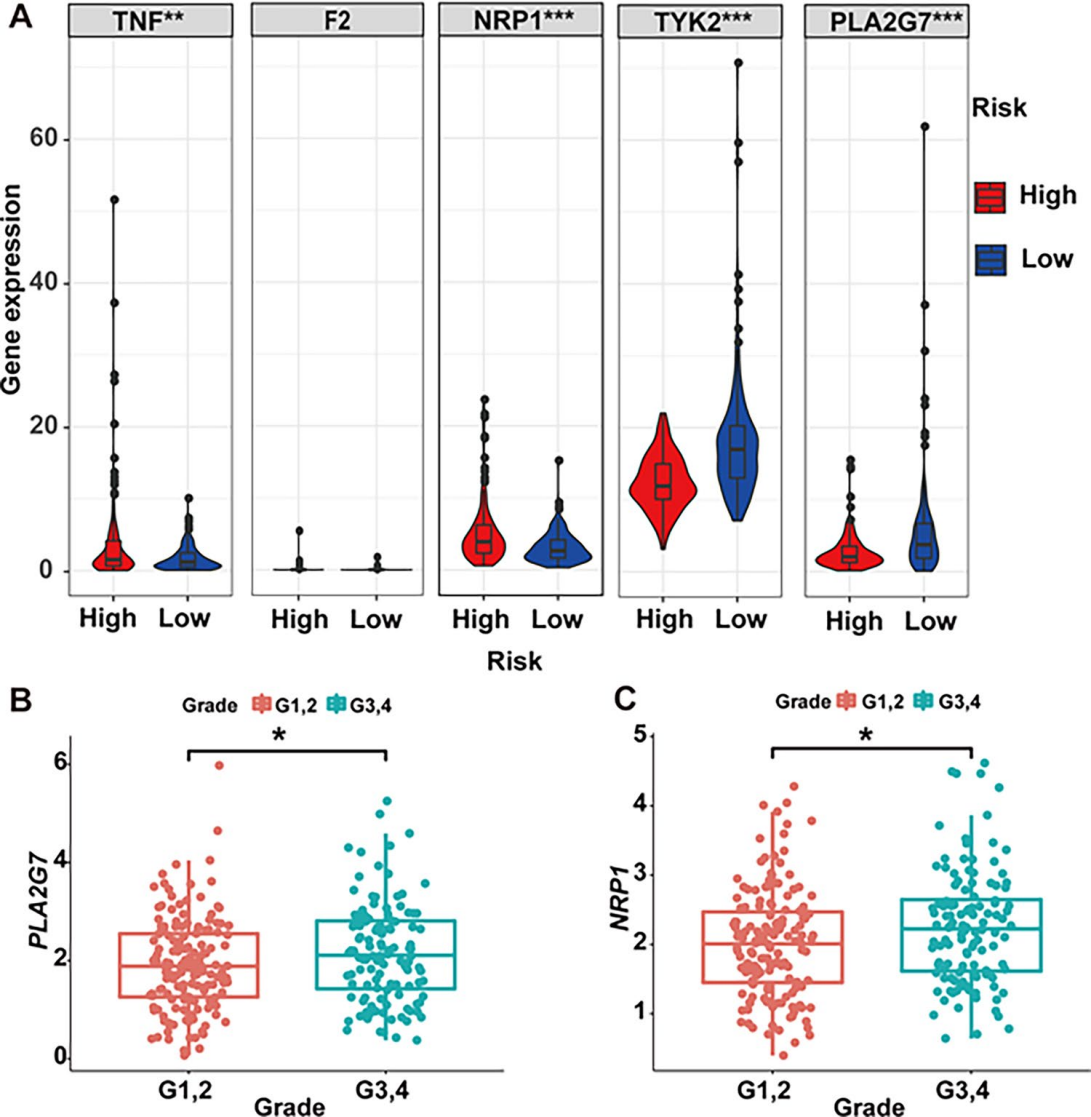
Differential expression of independent prognostic signatures. **A** Correlation between gene levels and risk scores. Expression levels of PLA2G7 (**B**), NRP1 (**C**), and their association with clinical characteristics. *P < 0.05; **P < 0.01; ***P < 0.001

Our exploration into overlapping genes’ impact on survival encompassed both univariate and multivariate Cox regression analyses. Eight genes emerged from univariate Cox regression, including TNF (tumor necrosis factor), IFNG (interferon gamma), IL1A (interleukin-1), F2 (coagulation factor II), NRP1 (neuropilin 1), TYK2 (tyrosine kinase 2), IDO1 (indoleamine 2,3-dioxygenase 1), and PLA2G7 (phospholipase A2 group VII) (*P* < 0.05, Fig. 4A). Subsequent multivariate Cox regression identified five independent prognostic factors-TNF, F2, NRP1, TYK2, and PLA2G7 (*P* < 0.05, Fig. 4B). The risk score was calculated as follows: risk score = (0.079934737 * *TNF* exp.) + (0.754296176 * *F2* exp.) + (0.077712628 * *NRP1* exp.) − (0.052768486 * *TYK2* exp.) − (0.130543819 * *PLA2G7* exp.). The derived risk score formula enabled the stratification of patients into high- and low-risk groups, with high-risk associated with poorer OS (Fig. 4C). ROC analysis showed robust predictive performance, with AUCs of 0.733, 0.738, and 0.614 at 3, 5, and 10 years, respectively (Fig. 4D). Survival scatter plots demonstrated the superior survival of low-risk patients (Fig. 4E, F). Notably, TNF, NRP1, TYK2, and PLA2G7 exhibited differential expression between high- and low-risk groups. The expression of TNF and NRP1 was lower in low-risk group, while the expression of TYK2 and PLA2G7 was higher in low-risk group (Fig. 5A). Additionally, NRP1 and PLA2G7 displayed heightened expression in grade 3/4 compared to grade 1/2 (Fig. 5A–C).

### Functional enrichment analyses

**Fig. 6:**
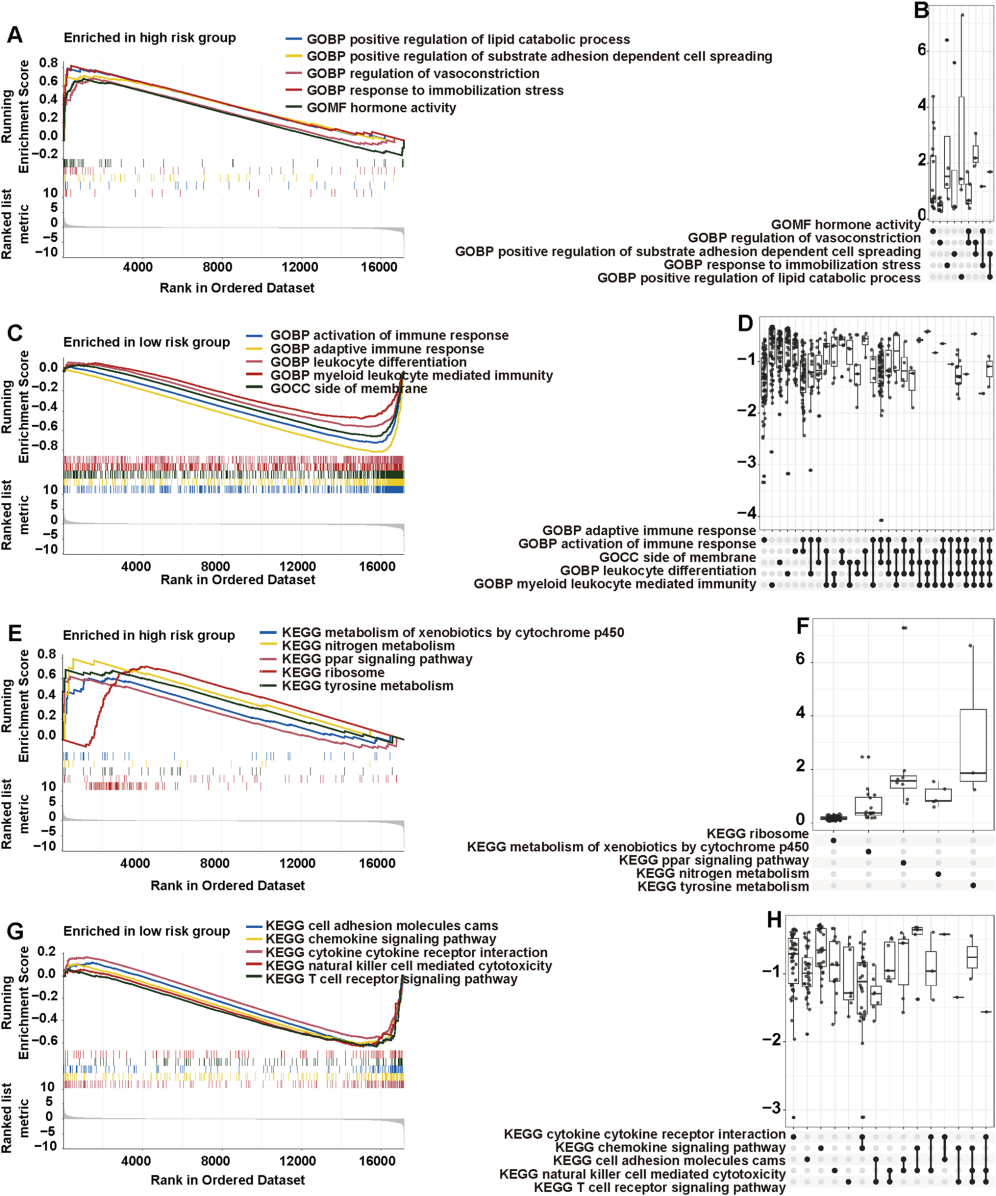
GO and KEGG Pathway Enrichment Analysis. Enrichment plot illustrating the running sum of enrichment scores (left curve), gene position within pathways (middle part), and metric distribution (bottom part) in high and low-risk groups. The upSet plot displays the metric distribution of core enrichment genes. **A**, **B** GO analysis in the high-risk group. **C**, **D** GO analysis in a low-risk group. **E**, **F** KEGG pathway enrichment in the high-risk group. **G**, **H** KEGG pathway enrichment in a low-risk group

The high-risk group was linked to responses to immobilization stress, lipid catabolic processes, substrate adhesion-dependent cell spreading, vasoconstriction, and hormone activity in the c5.go dataset (Fig. 6A, B). Conversely, the low-risk group exhibited significant enrichment of immune-related biological processes such as adaptive immune response, activation of the immune response, and myeloid leukocyte immunity (Fig. 6C, D). In the c2.cp.kegg dataset, the high-risk group displayed associations with ribosome, PPAR signaling pathway, tyrosine metabolism, nitrogen metabolism, and metabolism of xenobiotics by cytochrome P450 (Fig. 6E, F). On the other hand, the low-risk group was enriched in cytokine receptor interaction, chemokine signaling pathway, cell adhesion molecules, T cell receptor signaling pathway, and natural killer cell-mediated cytotoxicity (Fig. 6G, H).

### Tumor immune microenvironment and immunotherapeutic response

**Fig. 7:**
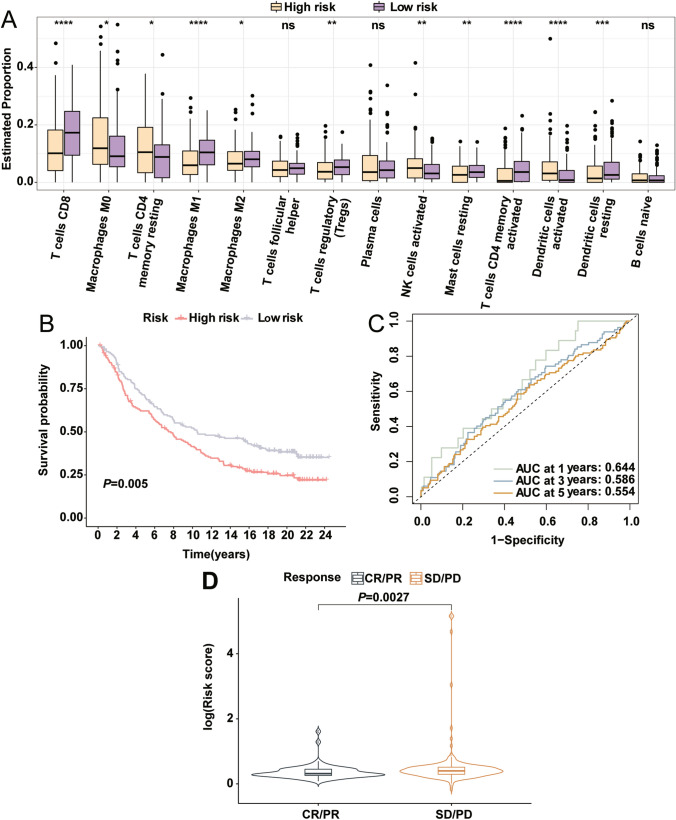
Immune Microenvironment and Therapeutic Response. **A** Comparison of immune cell infiltration between high-risk and low-risk groups. **B** Kaplan-Meier survival curves for IMvigor210 cohort based on risk scores. **C** ROC curves assessing predictive accuracy of the risk score in the IMvigor210 cohort. **D** Comparison of risk scores in different anti-PD-L1 treatment response groups. ****P < 0.0001, **P < 0.01, *P < 0.05

Application of CIBERSORT unveiled variations in the tumor immune microenvironment between high- and low-risk groups in CESC. The high-risk group exhibited lower immune cell infiltration levels, particularly CD8+ T cells, M1 macrophages, M2 macrophages, regulatory T cells (Tregs), resting mast cells, activated memory CD4+ T cells, and dendritic cells. Conversely, infiltration of M0 macrophages, resting memory CD4+ T cells, active NK cells, and dendritic cells was significantly elevated in the high-risk group (Fig. 7A). Employing the IMvigor210 immunotherapy cohort, we found that low-risk patients enjoyed notable survival benefits from anti-PD-L1 immunotherapy (*P* = 0.005) (Fig. 7B). The risk model demonstrated fair predictive accuracy for immunotherapy responsiveness with AUC values of 0.644, 0.586, and 0.554 at 1, 3, and 5 years, respectively (Fig. 7C). Further analysis established that low-risk patients were more responsive to immunotherapy (*P* = 0.0027) (Fig. 7D).

### Association of genes with tumor immune infiltrates

**Fig. 8:**
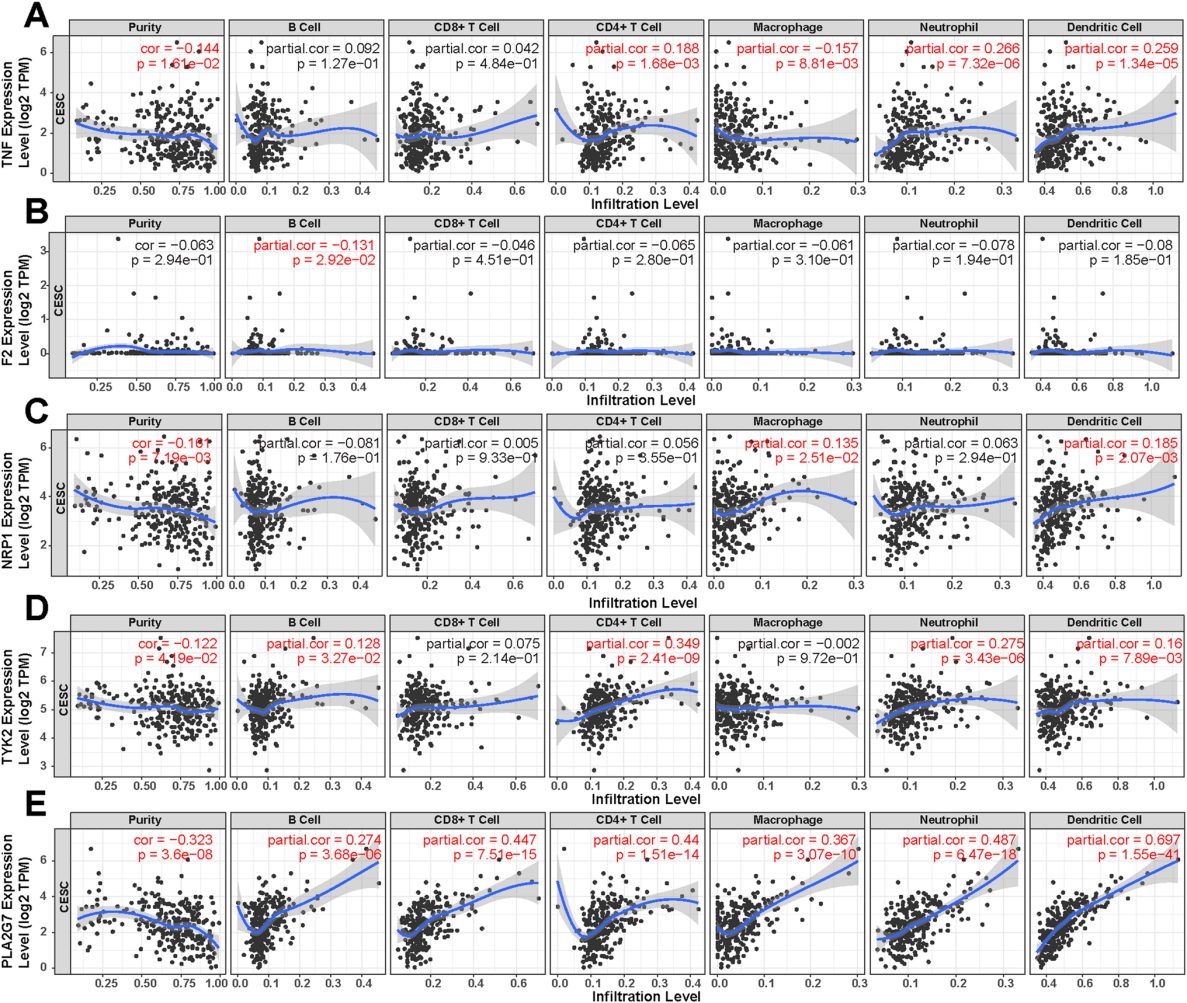
Correlation of Gene Expression with ESTIMATE Score. Correlation between the expression of each gene (**A: TNF; B, F2; C: NRP1; C: TYK2; E: PLA2G7**) and ESTIMATE Score in CESC.

**Fig. 9:**
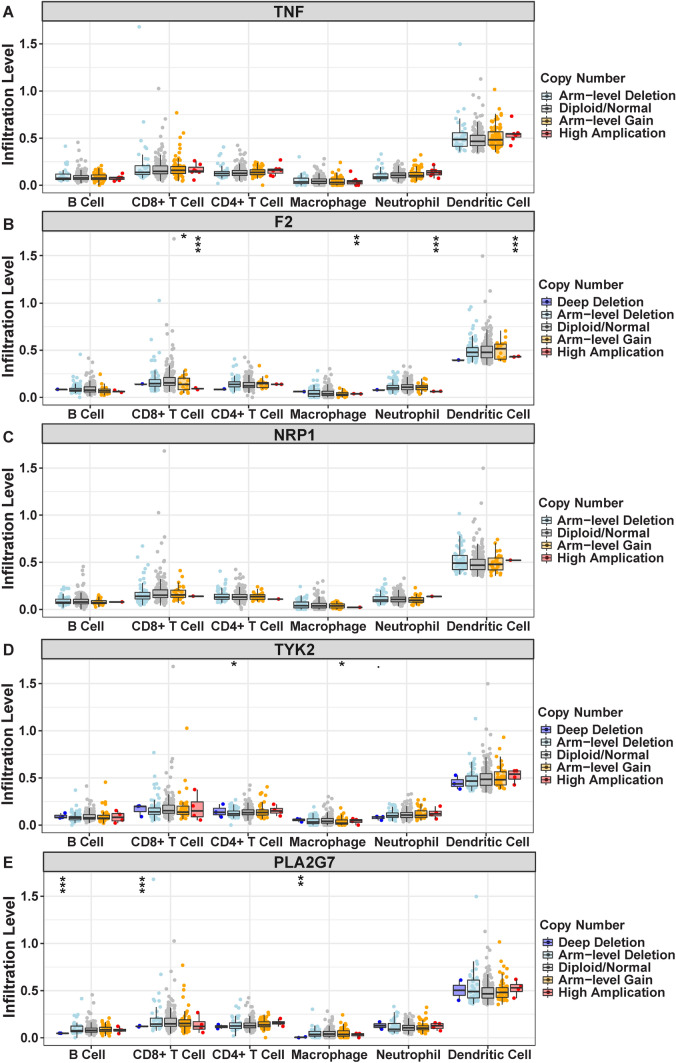
Effect of copy number variation on immune infiltration. Impact of copy number variation of each gene (**A: TNF; B: F2; C: NRP1; D: TYK2; E: PLA2G7**) on immune cell infiltration levels

Exploring the correlation between the expression of TNF, F2, NRP1, TYK2, and PLA2G7 and immune cell infiltrates revealed positive associations between higher expression levels and increased immune cell infiltration in tumors, including B cells, CD8+ T cells, CD4+ T cells, macrophages, neutrophils, and dendritic cells (Fig. 8A–E). Moreover, copy number variations (CNVs) of these genes exhibited notable impacts on immune cell infiltration levels. Specifically, deep deletion, arm-level deletion, arm-level gain, and high amplification of these genes significantly influenced immune cell infiltration in CESC (Fig. 9A–E).

### Docking results

**Fig. 10:**
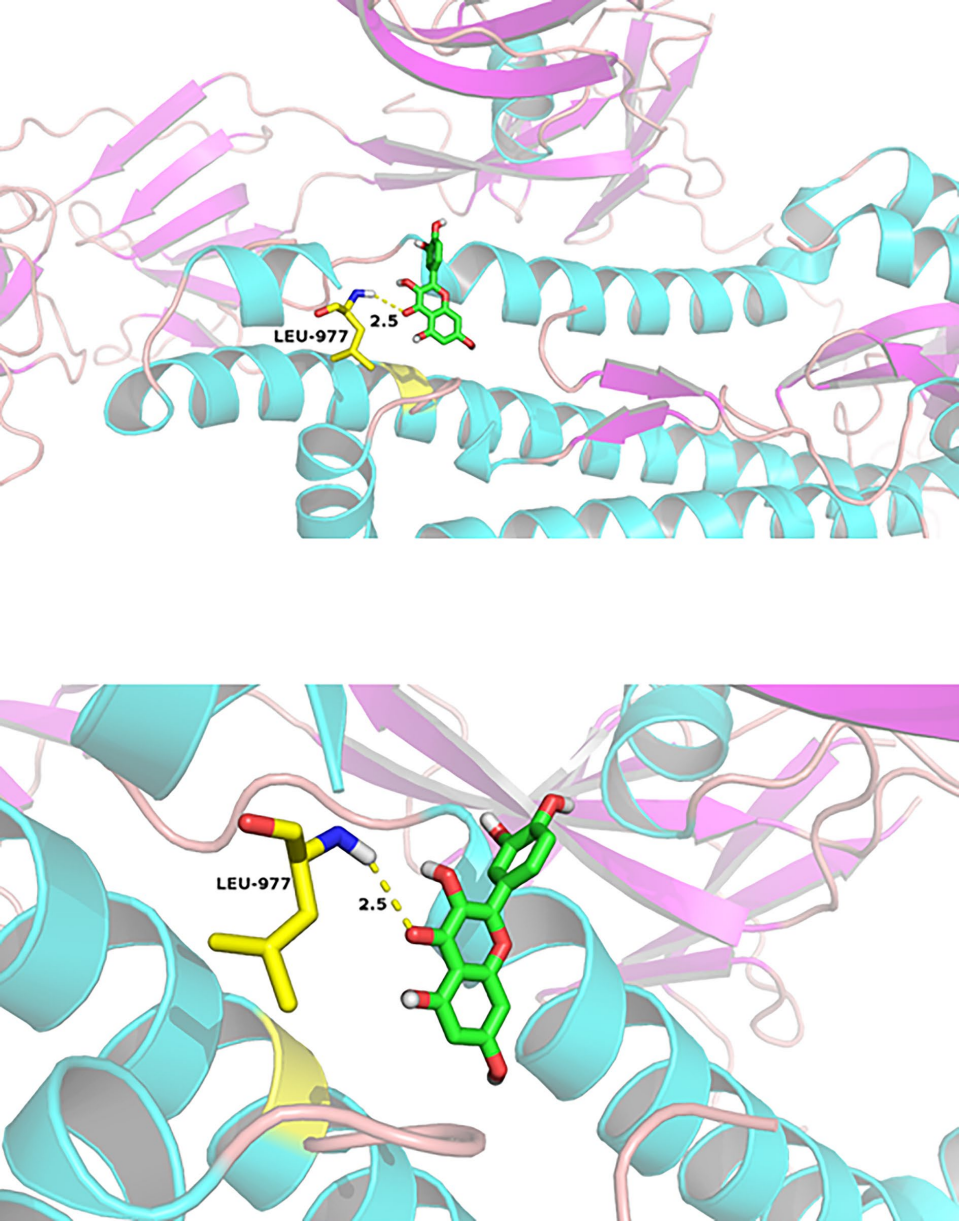
Molecular docking analysis. Diagram of quercetin binding to the spike glycoprotein of COVID-19 (PDB ID: 6VYB), assessed through molecular docking analysis

Molecular docking assays revealed robust binding interactions between quercetin and the crystal structures of COVID-19 main protease and spike glycoprotein, with favorable docking energies and hydrogen bond formation (− 5.88 kcal mol^−1^ for main protease and − 3.42 kcal mol^−1^ for spike glycoprotein). The quercetin formed hydrogen bonds with residues GLU-166 and THR-190 of the main protease and residue LEU-977 of spike glycoprotein (Table 1). Moreover, the docking analysis indicated favorable binding activities between quercetin and the five independent prognostic signatures with docking energy less than − 5 kcal mol^−1^ and two or more hydrogen bonds formed (Fig. 10; Table 2).

**Table 1:** Molecular docking results of quercetin with COVID-19 proteins

Protein	Docking score (kcal/mol)	Residues (hydrogen bond)
Main protease	− 5.88	GLU-166, THR-190
Spike glycoprotein	− 3.42	LEU-977

**Table 2:** Docking scores and hydrogen bonds of five independent prognostic signatures with quercetin

Gene name	Docking score (kcal/mol)	Number of hydrogen bonds
*TNF*	− 5.8	3
*F2*	− 6.5	3
*NRP1*	− 6.1	4
*TYK2*	− 7.1	3
*PLA2G7*	− 5.9	3

## Discussion

Evidence has shown potential therapeutic strategies for colon adenocarcinoma (COAD) and COVID-19 by quercetin. A total of 105 gene were potential targets of quercetin for anti-COAD/COVID-19, which were correlated with apoptosis, immune, oxidative stress. IL-17, HIF-1 and TNF were involved in quercetin-mediated treatment in COAD/COVID-19 patients [39]. Our investigation corroborates previous studies showcasing quercetin’s synergistic anti-CESC effects when combined with cisplatin [40, 41]. Employing network pharmacology, we pinpointed 45 target genes through which quercetin exerts its action against CESC and COVID-19. Enrichment analysis illuminated the therapeutic mechanisms, primarily centered around necroptosis, cytokine-cytokine receptor interaction, and viral protein interaction with cytokine and cytokine receptor pathways. Importantly, we identified that five independent prognostic signatures, including TNF, F2, NRP1, TYK2, and PLA2G7, via multivariate Cox regression could be biomarkers for prognosis.

*TNF* is a cytokine with potent pro-inflammatory effects [42]. Previous studies have shown that *TNF* had antitumor activity [43], playing an important role in tumor proliferation, migration, and invasion [44]. In CESC, it could induce apoptosis by activating the mitochondrial caspase-9 death signaling pathway. Moreover, recent study found that serum *TNF* level in patients with CESC were usually significantly elevated than this in non-CESC patients [45]. After surgical treatment, serum *TNF* level will gradually return to normal in CESC patients. Consistent with previous researches, *TNF* was considered as a risk factor for prognosis of CESC in our study. The expression level of *TNF* was significantly higher in high-risk group and tumor tissue than that in low-risk group and normal tissue.

Besides, current studies have proved that TNF-308 gene GA and AA polymorphisms were associated with the risk of cervical cancer [46, 47]. Moreover, TNF-308 AA and IL-10-592 CA/AA polymorphisms are linked to an increased risk of cervical cancer [48]. *F2* was considered as an important determinant of thrombin generation [49]. A number of studies discovered that complement activation following SARS-CoV-2 infections generates thrombin and produces thrombosis [50, 51]. *NRP1* is a semaphorin III receptor that is the foundation of neurosynapses [52]. *NRP1* has been reported to induce tumor-associated macrophage activation and exert pro-tumor effects in cervical cancer under hypoxia [53]. Previous study have shown that NRP1 was associated with cervical cancer progression and poor survival, suggesting that NRP1 could be an independent prognostic factor in cervical cancer [54]. High expression of NRP1 in cervical cancer patients was correlated with shorter OS [55]. Moreover, preoperative chemoradiation therapy reduced Treg and Nrp1 + Treg levesl in lymph nodes of cervical cancer patients [56]. Soluble NRP1 (sNRP) in circulating and NRP1 proteins were associated with cervical cancer stages. In addition, sNRP exhibited a possible diagnostic biomarker for cervical cancer [57]. Transportin-1 (TNPO1)-induced nuclear import of FUBP1 (Far upstream element binding protein 1) led to tumor immune evasion via upregulation of NRP1 in cervical cancer [58]. And high expression level of *NRP1* was associated with poor 5-year survival rates. Similarly, *NRP1* was confirmed as an oncogene in this study.

In COVID-19 patients, a significant decrease in *TYK2* level was observed in male patients compared to male controls [59]. The down-regulation of *TYK2* was proposed as a molecular mechanism causing SARS-CoV-2 to be incapable of inducing a competent interferon response. Castineira et al. [60] demonstrated that there is an association between life-threatening diseases in COVID-19 and high level of *TYK2*. *PLA2G7* is a calcium-independent lipoprotein-binding phospholipase, involved in cell signaling and metabolism [61]. On the one hand, some studies have shown that high level of *PLA2G7* was positively correlated with aggressiveness in cancer [62–64]. PLA2G7 promoted cell migration and invasion in prostate cancer [63]. Meanwhile, Morigny et al. demonstrated that secretion and expression of *PLA2G7* are positively correlated with cancer cachexia [65]. Depletion of PLA2G7 reduced intestinal polyposis and tumorigenesis in APC (Min/+) mice [66]. PLAG7 exhibited protective function in breast cancer via negative regulation of the Wnt signaling pathway [67]. On the other hand, Liao et al. discovered that high *PLA2G7* protein level was associated with significantly longer OS than low protein level of *PLA2G7* in ovarian cancer patients. The protective character of *PLA2G7* was speculated to be mediated by negatively regulating the Wnt/β-catenin pathway [68]. Similarly, our analysis showed that PLA2G7 was associated with a better prognosis in CESC.

Quercetin has been reported to influence SARS-CoV-2 infection and COVID-19-associated cancer progression via suppression of HIF-1a and mTOR [69]. Quercetin influenced several signaling pathways, including TNF, TRAIL and FASL, and induced cell apoptosis in cervical cancer [70]. There are several limitations in this study. For example, a comprehensive cohort of 306 CESC samples and 3 normal cervical tissues from TCGA was used. Only 3 normal cervical tissues were included in this study. In addition, this work lacks cell line experiments and animal studies. It is worthy to note that in vitro experiments and in vivo mouse models are necessary to validate the function of quercetin for the treatment of COVID-19 with CESC.

## Conclusion

Based on the results above, we believe that adjuvant therapy with quercetin may contribute to the treatment of COVID-19 or COVID-19 with CESC. The findings from this study identified molecules involved in the link between CESC and COVID-19, and five key genes of quercetin for CESC/COVID-19 treatment, including *PLA2G7*, *TNF*, *TYK2*, *F2*, *NRP1*. This finding can help the treatment of patients with CESC/COVID-19.

## Data Availability

The data are available from the corresponding author upon reasonable request. NCBI: https://www.ncbi.nlm.nih.gov/. GeneCards Database:https://www.genecards.org/. TCGA:https://portal.gdc.cancer.gov/. GTEx:https://www.gtexportal.org/. CTD: https://ctdbase.org/. DrugBank: https://go.drugbank.com/. SwissTargetPrediction: http://swisstargetprediction.ch/. TargetNet: http://targetnet.scbdd.com/. Kyoto Encyclopedia of Genes and Genomes: https://www.genome.jp/kegg/. g:Profiler: https://biit.cs.ut.ee/gprofiler. STRING: https://cn.string-db.org/. Protein Data Bank website: https://www.rcsb.org/search.
